# Focal MR-guided focused ultrasound treatment of localized low-risk prostate cancer

**DOI:** 10.1186/2050-5736-2-S1-A7

**Published:** 2014-12-10

**Authors:** Alexandr Nosov, Vladimir Turkevich, Christopher Cheng, Andrey Vorobiev, Sergey Kanaev, Sergey Reva, Georg Gafton

**Affiliations:** 1 Petrov Research Institute of Oncology, St. Petersburg, Russia

## Introduction

Focal magnetic resonance guided intensity focused ultrasound treatment offers a novel strategy that targets the cancer rather than the prostate in an attempt to preserve tissue and function. Its aim is to achieve long-term cancer control with minimal morbidity.

## Materials and methods

A prospective, ethics committee approved trial was conducted to determine the side effects of focal magnetic resonance guided intensity focused ultrasound treatment on ExAblate 2100 (InSightech). The purpose of this study was to evaluate the safety and initial effectiveness of focal ExAblate MRgFUS treatments for the treatment of organ-confined low risk prostate cancer (LRPC). 22 Adult males between the age of 49 and 73 were underwent 23 focal treatments for locally confined prostate cancer. One patient underwent two treatments due to two different foci.

## Results

5 SAE’s were reported; 4 of them were treatment-related. 3 treatment-related SAE’s were acut Urinary tract symptoms: Were reported in the questionnaires by 9 patients out of 23 (39%) during their last follow-up visit; 2 of them were reported as SAE’s. Urinary Incontinence: Was reported by 2 patien Biopsy Results after 6 months of follow-up are available for 23 patients. 2 patients dropped out of the study prior to completion of their follow-up periods; i.e., one patient - due to gradual continuous rise of PSA levels (with positive 6-mo biopsy in a newly detected focus) and the second patient due to moving to another country. At 6-mo follow-up 9 out of 23 biopsies (39%) were positive; all of them were in newly detected foci (from sectors that were negative at screening); 2 of these biopsies comprised stage upgrading as compared to baseline, (i.e., Gleason 7).

## Conclusions

Focal ExAblate treatment has a promising safety profile, however cancer localization improved is crucial for tumor control.

